# Emerging Genomic and Immunological Correlates Defining Oligometastatic Trajectories in Intermediate/High-Grade Soft-Tissue Sarcomas

**DOI:** 10.3390/genes17030323

**Published:** 2026-03-16

**Authors:** Alessandro Ottaiano, Francesco Sabbatino, Carmine Picone, Nadia Di Carluccio, Igino Simonetti, Annabella Di Mauro, Salvatore Tafuto

**Affiliations:** 1 Istituto Nazionale Tumori di Napoli, IRCCS “G. Pascale”, Via Mariano Semmola, 80131 Naples, Italy; c.picone@istitutotumori.na.it (C.P.); igino.simonetti@istitutotumori.na.it (I.S.); annabella.dimauro@istitutotumori.na.it (A.D.M.); s.tafuto@istitutotumori.na.it (S.T.); 2 Department of Medicine, Surgery, and Dentistry, University of Salerno, 84081 Baronissi, Italy; fsabbatino@unisa.it; 3 Department of Economics, University of Foggia, 71121 Foggia, Italy; nadia.dicarluccio@unifg.it

**Keywords:** soft-tissue sarcoma, oligometastatic disease, CINSARC, local ablative therapies, metastatic kinetics

## Abstract

Soft-tissue sarcomas (STSs) comprise a rare, heterogeneous group of mesenchymal malignancies in which histologic grade remains the strongest determinant of outcome, metastatic risk, and therapeutic strategy. Intermediate/high-grade STSs exhibit a pronounced propensity for early distant relapse, yet growing evidence indicates that metastatic behaviour is not uniform. Within this spectrum, an oligometastatic phenotype, characterised by a limited number of metastases, often confined to the lung, has emerged as a clinically and biologically distinct state associated with more indolent metastatic kinetics and improved survival when treated with aggressive local interventions. However, the criteria that define true oligometastatic STSs remain unsettled, and prospective evidence is lacking. Emerging molecular and immunological correlates provide a potential framework for biological triage. Low genomic complexity (low-risk CINSARC), a B-cell/TLS-rich tumour microenvironment, high immune-cytotoxic signatures, and persistently low or undetectable circulating tumour DNA (ctDNA) are each linked to reduced metastatic competence and may underpin oligometastatic trajectories. Conversely, high chromosomal instability, immunosuppressive microenvironments, and elevated ctDNA levels align with covertly polymetastatic biology despite limited radiographic disease. In this context, artificial intelligence and machinelearning approaches applied to computational genomics, immune profiling, imaging, and liquid-biopsy data offer a powerful strategy to integrate these multi-dimensional features and refine predictions of metastatic behaviour in STS. Oligometastatic STS therefore represents a biologically definable subset amenable to multimodal management integrating local ablative therapies, systemic agents, and immune-based strategies. Prospective, biomarker-stratified trials are needed to validate selection frameworks and optimise treatment sequencing in this evolving therapeutic space.

## 1. Introduction

Soft-tissue sarcomas (STSs) represent a highly heterogeneous family of malignant neoplasms of mesenchymal origin arising in soft connective tissues (including muscle, fat, fibrous tissue, peripheral nerve sheath, and vascular structures) and account for less than 1% of all adult malignancies (n ≈ 0.7%) [1,2]. In adults, the incidence is commonly estimated at approximately 1–2 cases per 100,000 individuals per year, although recent global data indicate modest increases in absolute case numbers with relatively stable or slightly declining age-standardised incidence rates [3,4], and a five-year overall survival of roughly 60% [1,5].A key feature of STS is the marked heterogeneity in histologic subtype, site of origin, biologic behaviour and clinical outcome; this makes generalised statements difficult and mandates subtype-specific and grade-specific considerations [6].

However, in clinical practice, the grading of STS is a critical determinant of prognosis and therapeutic strategy. The most widely adopted systems in adult STS are the French Federation of Cancer Centers Sarcoma Group (FNCLCC) (three-tier: G1, G2, G3) and the National Cancer Institute (NCI) system (also a three-tier scale), both of which integrate parameters such as tumour differentiation, mitotic count and necrosis [7,8]. Multiple studies confirm that histologic grade is perhaps the single strongest predictor of the risk of distant metastasis and overall survival in adult STS: for example, in one large series of 1240 non-metastatic adult STS patients, the 5-year metastasis-free survival was approximately 90.8% for grade 1, 71.4% for grade 2 and 43.5% for grade 3 tumours [9]. Such data underscore why the distinction between low, intermediate and high grade is more than semantic, it drives risk stratification, surgical planning, decision-making about adjuvant therapy, and follow-up intensity [8,9]. Indeed, high-grade STSs (generally G2–G3 in FNCLCC terminology) are associated with a substantially elevated risk of metastatic spread (often to lung, but also to bone, liver or other sites), increased propensity for local recurrence, and markedly shortened overall survival compared to low-grade lesions [10].

Epidemiologically, STSs manifest across the adult age spectrum but with increasing incidence in older age groups; for instance, an age-standardised incidence rate (ASIR) of 6.34 per million person-years was reported in a large population-based study of extremity STSs, with peaks above age 65 years [11]. Still, variations by histologic subtype, tumour size, depth, site (extremity versus trunk/retroperitoneum) and grade complicate the picture and contribute to the observed heterogeneity in survival outcomes [12,13]. The rarity and morphological diversity of STS pose clinical challenges including delays in diagnosis, sub-optimal referral to specialised sarcoma centres, and variation in treatment pathways [1,12]. The evolution of disease in high-grade STS often follows a pattern of first local control (via wide-margin surgery ± radiotherapy) and then the risk of distant relapse, frequently within the first 2–3 years after diagnosis of the primary tumour [14]. For example, in a historical population-based Swedish series of 508 adult STSs of the extremity and trunk wall, the local-recurrence rate was approximately 30%, and most metastases developed within three years of primary treatment [15], a recurrence pattern that remains broadly similar today. Taken together, these epidemiologic patterns, risk-stratification principles, and early metastatic trajectories in high-grade STS underscore the need to refine our understanding of how metastatic spread unfolds across different biological subgroups, thereby setting the stage for a more nuanced exploration of metastatic phenotypes.

## 2. The Oligometastatic Paradigm in High-Grade STS

**Figure 1:**
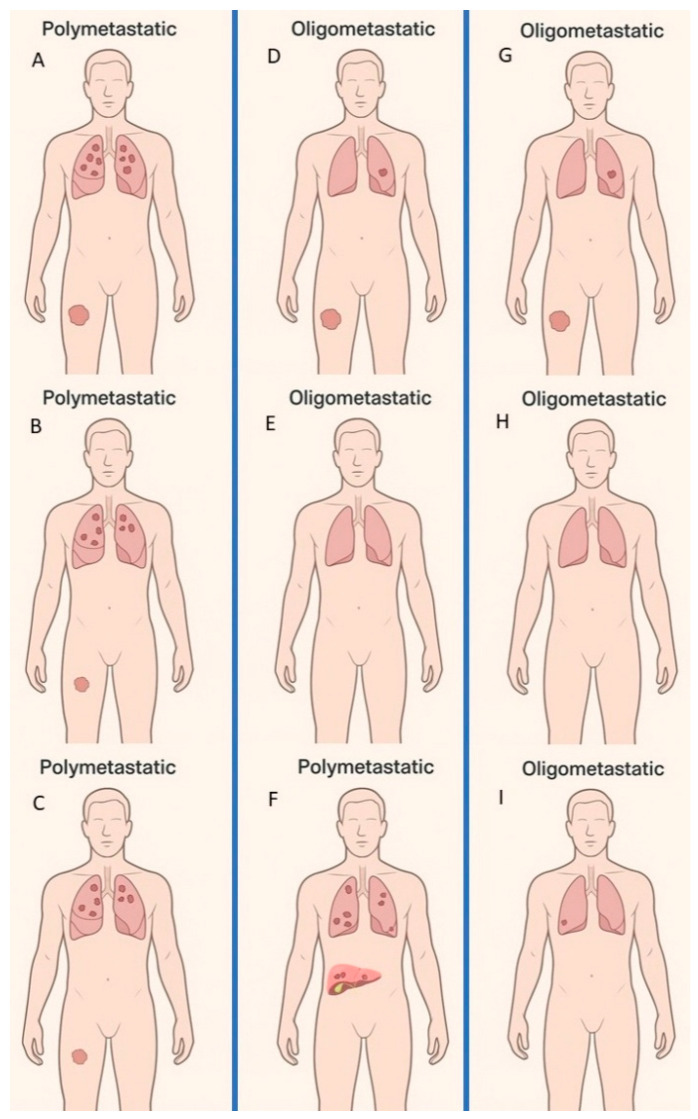
Clinical trajectories of polymetastatic, apparent oligometastatic, and true oligometastatic disease. Panel (**A**) illustrates a patient presenting with a high-grade extremity STS and multiple synchronous pulmonary metastases (polymetastatic disease). Standard management consists of front-line systemic therapy. Panels (**B**,**C**) show subsequent disease assessments demonstrating maintained disease control under systemic treatment. This scenario reflects a clinical setting in which systemic therapies—tailored to performance status, comorbidities, and patient goals—remain the principal therapeutic modality. Panel (**D**) depicts a patient with an extremity STS and a single pulmonary metastasis (apparent oligometastatic disease). Management includes radical resection of both the primary tumour and the metastatic lesion, followed by adjuvant chemotherapy depending on histology. Postoperative reassessment in Panel (**E**) shows no evidence of disease (NED). However, at subsequent follow-up (Panel (**F**)) the patient develops widespread polymetastatic relapse with multiple pulmonary and hepatic lesions, revealing an underlying biologically polymetastatic phenotype despite the initial oligometastatic presentation. Panel (**G**) shows a patient with an analogous clinical presentation to Panel (**D**) (extremity STS with a single pulmonary metastasis) managed with radical surgical treatment. After a period of NED, the patient develops an indolent, limited recurrence pattern (Panel (**H**,**I**)), manifesting as a single new pulmonary metastasis (oligo-recurrence). Despite similar initial presentations, patients in Panels (**D**,**G**) exhibit fundamentally distinct evolutionary trajectories, distinguishing pseudo-oligometastatic from true oligometastatic disease.

Within the metastatic spectrum of STS, the concept of oligometastatic versus polymetastatic disease has recently gained increasing interest (Figure 1). The term “oligometastatic” describes a state of limited metastatic dissemination, commonly defined in solid tumours as up to five discrete metastatic lesions, often confined to one or two organs, with the possibility of curative or long-term durable local therapy [16]. Although originally proposed in carcinomas, such as colorectal or lung cancer, this paradigm is increasingly applied to STSs [17]. Indeed, retrospective analyses in STSs suggest that a subgroup of patients with limited metastatic burden may experience prolonged survival or even long-term disease control when treated with aggressive local therapies (surgery, stereotactic radiotherapy, ablation) in combination with systemic therapy, with 5-year overall-survival rates up to 50% [17,18].

The interest in distinguishing oligo- from polymetastatic disease in high-grade STS arises from both biological and therapeutic perspectives. Biologically, the oligometastatic state may reflect a less aggressive cell clone, slower metastatic kinetics, possibly limited metastatic competence or effective host immunesurveillance; this situation may open a window for interventions aimed at long-term control rather than purely palliative intent. From a therapeutic standpoint, identifying patients with intermediate/high-grade STSs who manifest a clinically indolent or pauci-diffuse metastatic biology (oligometastatic) offers the opportunity to apply local ablative therapies (metastasectomy and stereotactic body radiotherapy) to potentially extend the progression-free interval, defer systemic therapy, and improve quality of life and survival [17,19]. Emerging evidence in STS research supports this approach: in cases treated with stereotactic body radiotherapy (SBRT) for ≤3 metastases, long median overall survival of 69 months was reported [20]. However, the boundaries of this oligometastatic state in STSs are neither definitively defined (regarding number of lesions, organs involved, timing relative to primary diagnosis) nor universally validated in prospective studies, which leaves considerable uncertainty in clinical decision-making [17,21].

In the context of intermediate/high-grade STSs, the exploration of oligometastatic trajectories is particularly pertinent. High-grade STSs represent patients at significant risk of metastatic progression; within this high-risk population, identifying those whose metastatic evolution remains relatively indolent (oligometastatic, pauci-diffuse) might allow stratification and tailoring of interventional therapy, rather than defaulting to systemic palliative chemotherapy alone. This holds particular relevance in designing treatment strategies that integrate local ablative therapy, immune or targeted adjuncts, and surveillance approaches.

In sum, while STSs are rare, heterogeneous and often aggressive, the evolving recognition of oligometastatic phenotypes within intermediate/high-grade disease invites a shift from uniform palliative metastatic paradigms toward potential durable control in a selected subset of patients. This review will therefore focus on intermediate/high-grade STSs, the clinical evolution of oligometastatic disease in this setting, and the therapeutic and translational implications thereof.

## 3. Biological Underpinnings of Oligometastatic Evolution in Intermediate/High-Grade STS

**Figure 2:**
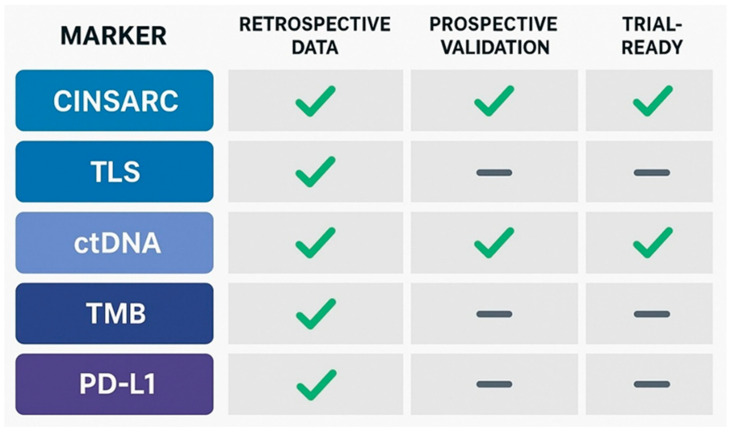
Evidence matrix of candidate biomarkers for differentiating oligometastatic from polymetastatic disease in soft-tissue sarcomas.

Molecular correlates underpinning an indolent, pauci-metastatic course in intermediate- and high-grade STS remain insufficiently characterised. However, several features have emerged as candidate biomarkers and biological programmes that are mechanistically plausible and supported by existing clinical and translational evidence (Figure 2).

First, low genomic complexity/low mitotic–chromosomal instability signatures (low-risk CINSARC) identify sarcomas with reduced propensity to metastasize: the Complexity INdex in SARComas (CINSARC) transcriptomic signature (composed of genes related to mitosis and chromosome maintenance) robustly stratifies metastatic risk across multiple STS cohorts, and low-risk CINSARC profiles are associated with markedly lower metastatic rates compared with high-risk profiles [22,23]. The CINSARC score is derived from the expression profile of 67 genes primarily involved in mitosis, chromosomal segregation, and maintenance of genomic stability. To calculate it, gene expression data (from microarray or RNA-seq) are normalised and compared to a reference expression matrix to assess relative transcript abundance for each CINSARC gene. Using the nearest-centroid classification method, each tumour sample is assigned to one of two transcriptional classes, C1 (low-risk) or C2 (high-risk), based on its overall correlation pattern with predefined centroids established from training sarcoma datasets. Within the oligometastatic conceptual framework, tumours classified as CINSARC-low (or equivalently with low measures of chromosomal instability) are the most plausible molecular substrate for limited seeding events and slow outgrowth of metastases. Clinical trials are now prospectively using CINSARC to enrich for high-risk patients for adjuvant systemic therapy, illustrating the signature’s translational maturity and its specific link to metastatic potential [24].

Second, tertiary lymphoid structures (TLS) and a B-cell-rich immune microenvironment have emerged as reproducible, clinically relevant predictors of favourable outcomes in STS and of enhanced response to immunotherapy. In a large, integrative analysis of STS immune classes, a distinct “B-cell/TLS-rich” immune class showed superior overall survival and higher response rates to PD-1 blockade; the presence of TLS correlates with organised local antigen presentation, intratumoural B-cell maturation and local adaptive immunity that plausibly restrains metastatic outgrowth and promotes local control of micrometastases [25,26]. Thus, TLS positivity/high B-cell signature (and related ICR/immune-active transcriptomic scores) constitute candidate biomarkers that identify a tumour microenvironment more capable of limiting dissemination or controlling microscopic deposits—a likely biological determinant of oligometastatic behaviour [27].

Third, measures of circulating tumour burden (ctDNA) and minimal residual disease (MRD) provide dynamic, patient-specific readouts that associate closely with metastatic burden and outcome. Multiple recent series and reviews in sarcoma show that detectable/quantifiable ctDNA correlates with radiographic disease burden and worse outcome, whereas undetectable or very low ctDNA after definitive therapy is associated with lower relapse risk and longer disease-free intervals [28,29]. Translationally, patients with a clinical phenotype of limited metastases and persistently low/absent ctDNA are biologically distinct from those with high ctDNA allele fractions and rapidly progressive, multifocal disease; therefore, ctDNA negativity (or low fractional abundance) is a practical biomarker to enrich for oligometastatic candidates and to monitor for progression after metastasis-directed therapy.

Fourth, tumour mutational burden (TMB), neoantigen load and specific driver lesions modulate immune visibility and metastatic competence. Although most STS types are classically low-TMB, heterogeneity exists: certain complex-karyotype entities (e.g., undifferentiated pleomorphic sarcoma, some pleomorphic liposarcomas) may harbour higher TMB and more neoantigens, which can both increase the probability of immune recognition and, paradoxically, reflect genomic instability linked to aggressive behaviour [30,31]. In practice, low TMB in the context of immune-active microenvironments (high TLS/ICR) appears more consistent with restrained metastatic progression, whereas very high genomic instability/TMB often reflects aggressive biology with greater seeding potential. Thus, the combination of TMB/genomic complexity and immune contexture (not either alone) best predicts metastatic evolution.

Fifth, immune effector metrics beyond TLS, CD8+ T-cell infiltration, T-cell clonality and PD-L1 expression, are variably associated with outcome in STS and may refine prediction. Several translational reports show that robust T-cell infiltration and clonality correlate with immune checkpoint expression and, in some subtypes, with improved outcomes or immunotherapy responsiveness [32]. PD-L1 expression alone is an inconsistent predictor in STS, but when interpreted together with T-cell density, clonality and B-cell/TLS presence it contributes to a composite picture: tumours with coordinated adaptive immune responses (high ICR/TLS/CD8 clonality) are biologically more capable of suppressing metastatic outgrowth [26,32].

Finally, specific genomic drivers and tumour lineage still matter: translocation-driven sarcomas (e.g., synovial sarcoma, myxoid liposarcoma) often have more predictable metastatic tropisms and, in some cases, protracted natural histories with late or site-restricted metastases, while complex-karyotype sarcomas (UPS, dedifferentiated liposarcoma) more frequently follow polymetastatic trajectories [33]. Large contemporary genomic surveys confirm that histology-specific genomic landscapes (*TP53* alterations, *MDM2*/*CDK4* amplification, fusion oncoproteins) interact with immune and transcriptomic programmes to shape metastatic competence [30]. Taken together, the most evidence-backed, actionable molecular predictors of an oligometastatic course in intermediate/high-grade STS are: (a) low CINSARC (low genomic complexity); (b) presence of B-cell/TLS-rich immune microenvironment and high ICR/immune-active transcriptomic scores; (c) persistently low or undetectable ctDNA (MRD negativity) after definitive therapy; and (d) a genomic profile with limited copy-number chaos and moderate/low TMB in the context of immune activation. Importantly, the predictive power may increase when these variables are combined into multimodal classifiers (genomic + immune + circulating markers) rather than used singly. Prospectively validating composite biomarker models and integrating them into trials of metastasis-directed therapy (surgical metastasectomy, SBRT ± ICIs—immune checkpoint inhibitors) represents the next translational imperative to move from retrospective associations to clinically actionable patient selection.

Transitioning from retrospective associations to prospectively validated, biomarker-driven patient selection constitutes a major translational challenge. The principal barriers are predominantly methodological and organisational rather than conceptual. First, STSs are rare and biologically heterogeneous, rendering adequately powered, biomarker-stratified prospective trials difficult to design and slow to accrue. Second, most candidate biomarkers discussed in our manuscript (e.g., CINSARC, TLS/B-cell signatures, ctDNA dynamics) have been validated primarily in retrospective or exploratory translational cohorts, frequently using different analytical platforms and cut-offs; thus, analytical standardisation and cross-platform harmonisation are prerequisites for routine clinical implementation. Third, composite models integrating genomics, immune profiling, and liquid-biopsy data require harmonised workflows, centralised testing facilities, and robust bioinformatics infrastructures—resources that are not uniformly available across institutions. Finally, in the oligometastatic setting, randomising patients between metastasis-directed therapy and alternative strategies raises ethical and practical challenges, particularly in the absence of clear clinical equipoise. Nonetheless, we consider the prospects for progress realistic in the near- to mid-term, particularly within collaborative sarcoma networks and high-volume referral centres.

## 4. Clinical Prognostic Tools and Nomograms Relevant to Metastatic Risk

Beyond histologic grade and classical clinicopathologic variables, several validated prognostic instruments provide refined estimates of metastatic risk, tumour burden, and overall survival in STSs, offering potential utility in distinguishing oligometastatic from polymetastatic trajectories. It is important to emphasise that these clinical risk models do not directly define oligometastatic disease, but they quantify the underlying metastatic competence and systemic risk that shape the probability of limited versus widespread dissemination, complementing emerging molecular biomarkers such as CINSARC, immune signatures and ctDNA.

Sarculator (www.sarculator.com, last accessed on 28 November 2025) is a widely used, externally validated prognostic nomogram originally developed by the Istituto Nazionale dei Tumori (Milan). It integrates clinical parameters (including age, tumour size, tumour depth, histologic subtype, and FNCLCC grade) to compute individualised predictions of overall survival and metastasis-free survival in localised STSs (sarcoma nomogram) [34,35]. Sarculator has been validated across multiple independent cohorts and consistently outperforms traditional AJCC staging in discriminating risk categories [36]. Because its predictive outputs correlate with metastatic potential and tumour dissemination kinetics, Sarculator may help identify high-grade STS patients with intrinsically low metastatic propensity who could align more closely with an oligometastatic biological phenotype. Conversely, high Sarculator-predicted risk supports early systemic therapy prioritisation consistent with polymetastatic biology.

A second widely adopted prognostic instrument is the Memorial Sloan Kettering Cancer Center (MSKCC) postoperative nomogram for extremity STSs, which incorporates tumour size, grade, depth, histology, surgical margins and patient age to estimate 12-year sarcoma-specific survival [37]. This nomogram has been validated both internally and externally and has demonstrated robust prognostic performance across diverse histologic subtypes [38]. Similar to Sarculator, the MSKCC tool provides insight into patient-specific metastatic risk and long-term prognosis, thereby contributing to clinical decision-making relevant to identifying candidates for metastasis-directed therapy.

Another clinically validated prognostic instrument increasingly adopted in STSs is PERSARC (Personalised Sarcoma Care). PERSARC is an evidence-based predictive nomogram developed by the Dutch Soft-Tissue Sarcoma Group to estimate individualised survival probabilities and treatment-specific local-recurrence risks using routinely available clinical variables (histologic subtype, FNCLCC grade, tumour size, depth, and patient age) [39]. Unlike traditional static nomograms, PERSARC incorporates competing-risk methodology, allowing separate and dynamic predictions of overall survival, disease-specific survival, and local-recurrence risk for different therapeutic strategies (e.g., surgery alone versus surgery plus radiotherapy). This makes PERSARC particularly useful in multidisciplinary decision-making, especially for intermediate/high-grade tumours where balancing surgical morbidity, radiotherapy indications, and long-term functional outcomes is complex [40]. Early validation studies have shown that PERSARC provides accurate prognostic discrimination across extremity and trunk wall STS and improves concordance between recommended and observed treatment strategies [39,40].

## 5. Putative Genetic Drivers of Oligometastatic Versus PolyMetastatic Behaviour in Intermediate/High-Grade STS

Beyond global measures of genomic complexity and immune contexture, relatively little is definitively known about the specific genetic determinants that distinguish oligometastatic from polymetastatic trajectories in intermediate/high-grade STS. Nevertheless, emerging evidence suggests that discrete driver alterations may contribute to divergent metastatic behaviours. Complex-karyotype sarcomas—such as undifferentiated pleomorphic sarcoma (UPS) and dedifferentiated liposarcoma—are frequently characterised by alterations in *TP53* and *RB1* and amplifications of *MDM2*/*CDK4*, leading to impaired cell-cycle checkpoints, defective DNA-damage responses, and permissive chromosomal instability. Comprehensive genomic profiling studies have shown that *TP53* pathway disruption correlates with higher copy-number burden and increased metastatic propensity, supporting the hypothesis that genomic instability may facilitate early dissemination and polymetastatic spread [22]. By contrast, tumours retaining partial genomic integrity or lacking high-level chromosomal instability may undergo slower clonal diversification, potentially constraining metastatic seeding to a limited number of sites.

Translocation-driven sarcomas represent a biologically distinct paradigm. Entities such as synovial sarcoma (*SS18*–*SSX* fusions) and myxoid liposarcoma (*FUS*–*DDIT3*) are genomically “simple,” typically exhibiting low tumour mutational burden (TMB) and relatively stable karyotypes [41,42]. Although clearly capable of metastatic spread, their dissemination may follow more predictable patterns and, in selected cases, longer intervals before widespread systemic progression. The relative genomic simplicity of fusion-driven sarcomas could reduce the probability of acquiring additional prometastatic driver events compared with complex-karyotype counterparts. However, secondary alterations—such as *TP53* mutations in synovial sarcoma or activation of the PI3K/AKT pathway—may accelerate progression, suggesting that the acquisition of cooperating drivers might represent a molecular shift from indolent/oligometastatic to aggressive/polymetastatic behaviour [43].

Alterations in cell-cycle regulators and mitotic control genes, central to the CINSARC signature, further support this conceptual framework. Overexpression or amplification of genes governing chromosomal segregation (e.g., *AURKA*, *BUB1B*, *CDC20*) has been associated with metastatic competence and adverse survival outcomes [22]. Mechanistically, such alterations may enhance proliferative velocity and genomic instability, facilitating clonal evolution and adaptation at distant sites. Conversely, tumours with lower expression of mitotic drivers might sustain more limited proliferative expansion at metastatic niches, a feature potentially compatible with an oligometastatic phenotype.

Epigenetic modifiers may also contribute to metastatic divergence. Dysregulation of *EZH2*, frequently overexpressed in aggressive STS, promotes chromatin remodelling that can favour invasion and immune evasion. Preclinical and translational data suggest that elevated EZH2 activity may correlate with metastatic progression and immunosuppressive microenvironments [44]. Similarly, aberrant activation of PI3K/AKT/mTOR signalling—through mutations in *PIK3CA* or loss of *PTEN*—has been associated with enhanced survival signalling and increased metastatic fitness [45,46].

While these associations remain largely correlative, they support a working model in which cumulative genetic and epigenetic alterations may progressively enable systemic dissemination, whereas their relative absence or limited acquisition could underlie more restrained metastatic trajectories.

## 6. Treatment of Oligometastatic STSs

Treatment of metastatic intermediate- and high-grade STSs must be individualised according to both histology and the distribution of metastatic disease, as therapeutic objectives and evidence differ substantially between polymetastatic and oligometastatic settings. In polymetastatic STS, systemic cytotoxic therapy remains the cornerstone of management. First-line anthracycline-based regimens (doxorubicin with or without ifosfamide) continue to represent the international standard. Randomised phase III trials have demonstrated higher response rates and improved progression-free survival with combination therapy; however, these benefits have not translated into a clear overall-survival advantage and are accompanied by significantly increased toxicity [47]. Beyond anthracyclines, approved or guideline-recommended systemic agents for advanced STSs include the multi-kinase inhibitor pazopanib (PALETTE: median PFS 4.6 vs. 1.6 months for placebo) and histology-directed agents (e.g., trabectedin, eribulin for selected subtypes), but median survival in unselected polymetastatic cohorts typically remains limited (median OS in contemporary series commonly in the range of ~12–20 months) [48,49].

By contrast, management of oligometastatic STSs, typically defined pragmatically as a small number (commonly ≤3–5) of metastases, often confined to a single organ (most frequently the lung) and/or a long disease-free interval (DFI), emphasises an aggressive, multimodal approach directed at long-term disease control. Surgical metastasectomy for pulmonary metastases has the most extensive historical evidence: large series and systematic reviews report that complete resection (R0) is associated with markedly improved outcomes compared with non-operative management, with reported 5-year overall survival after complete pulmonary metastasectomy ranging roughly from ~25% to >50% in selected series and longer median survival when selection criteria (single or few metastases, prolonged DFI, good performance status) are met [50,51,52]. Nevertheless, these findings derive primarily from observational and retrospective studies and are therefore vulnerable to selection bias. In the absence of randomised prospective trials comparing metastasectomy with non-operative approaches in STS, the observed survival benefit should be interpreted cautiously. Realistic prospects for addressing this gap may rely on the development of international, multicentre collaborative trials within established sarcoma networks, thereby enabling adequate patient accrual despite the rarity of the disease. Moreover, adaptive, registry-based, or platform trial designs could enhance feasibility while maintaining methodological rigour, ultimately facilitating prospective validation in this rare and biologically complex clinical setting.

When surgery is not feasible or to preserve organ function, local ablative therapies provide effective alternatives. Stereotactic body radiotherapy (SBRT) applied to oligometastatic STS lesions is the most commonly used ablative technique and has demonstrated high local control rates with favourable survival outcomes across several retrospective cohorts with local control at 2–4 years often >70–80% and median overall-survival estimates for selected oligometastatic cohorts of 49–69 months [53,54,55,56,57,58]. A pooled meta-analysis of SABR/SBRT in oligometastatic cancer across tumour types showed acceptable toxicity and clinically meaningful local control and survival outcomes in prospective studies, supporting SBRT as a safe, repeatable local modality for oligometastatic sarcoma patients [59]. Thermal ablation (radiofrequency or microwave) has also demonstrated high local-control rates for small pulmonary lesions from sarcoma, with institutional series reporting safe use and durable control in appropriately sized metastases and when combined with surgery in hybrid approaches [60,61]. In clinical practice, multimodal approaches combining resection, ablation and SBRT are increasingly used to treat sequential recurrences, with the explicit aim of extending systemic therapy-free intervals or achieving prolonged disease control.

However, the integration and sequencing of local and systemic therapies is typically decided within a multidisciplinary sarcoma centre. Pragmatic strategies include (i) upfront local therapy for clearly resectable/ablative limited disease with curative intent in patients with favourable clinical features (long DFI, ≤3 lesions, good performance status); (ii) peri-operative systemic therapy for high-risk histologies or when occult micrometastatic disease is suspected; and (iii) SBRT/ablation to defer systemic therapy when local control is achievable with low morbidity. Data supporting definitive rules for sequencing are limited and largely retrospective; therefore, treatment decisions remain individualised and evidence gaps motivate prospective studies.

Finally, minimally invasive molecular tools are rapidly transforming patient selection and post-treatment monitoring in the oligometastatic setting. As previously noted, ctDNA assays in sarcomas demonstrate promising concordance with radiographic tumour burden and carry significant prognostic value [28,29]. One of the clinical contexts in which ctDNA may prove particularly useful is the assessment of treatment efficacy following local ablative interventions. Detectable or rising ctDNA after definitive local therapy is associated with early relapse and poorer outcomes, whereas undetectable ctDNA, reflecting a state of molecular remission, is linked to a reduced risk of recurrence and may help identify suitable candidates for metastasis-directed approaches or for surveillance in the absence of immediate systemic therapy [28,29,62].

**Figure 3:**
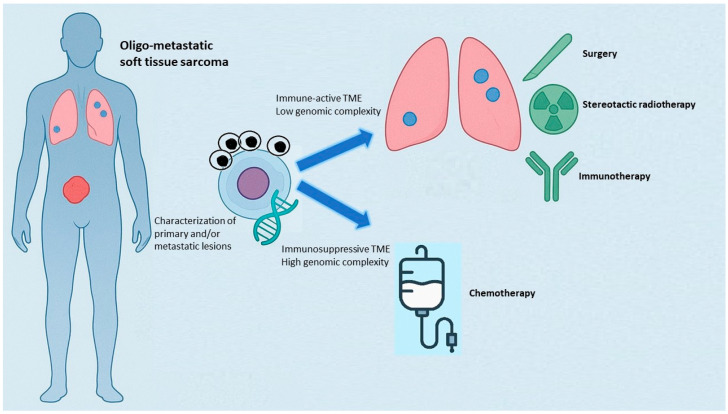
Conceptual framework for the biological triage of a patient with newly diagnosed oligometastatic STS. The diagram depicts a patient presenting with limited metastatic disease who undergoes molecular characterisation of the primary tumour and/or metastatic sites. This integrated characterisation diverges into two distinct evolutionary scenarios. In the first, a tumour with an immune-active microenvironment and low genomic complexity is interpreted as a biologically oligometastatic phenotype, more likely to benefit from radical local therapies in combination with immunotherapy. In the second, the profiling reveals an immunosuppressive tumour microenvironment (TME) coupled with high genomic complexity, indicating a covertly polymetastatic biology. In this setting, upfront systemic chemotherapy is favoured to address early disseminated potential. The figure illustrates how a biologically informed assessment provides a decision compass, guiding whether an ostensibly oligometastatic presentation should be approached with local radical intent or treated as a systemically evolving disease.

In summary, while systemic chemotherapy remains the cornerstone for polymetastatic high-grade STS (with modest median PFS/OS in unselected patients), the oligometastatic subset is increasingly managed with curative-intent local interventions (metastasectomy, SBRT, and percutaneous ablation) alone or integrated with systemic therapy. The current evidence is largely retrospective but indicates meaningful long-term survival and local-control rates in selected patients; prospective, biomarker-stratified trials (including ctDNA-guided algorithms) are urgently needed to standardise selection, sequence treatment modalities and quantify the incremental benefit attributable to metastasis-directed interventions (Figure 3).

## 7. Emerging Innovative Translational and Immunotherapeutic Strategies in Oligometastatic STS

In the evolving landscape of STS, the oligometastatic state offers a compelling translational opportunity for the incorporation of immune- and target-based therapies alongside local ablative approaches. Recent reviews highlight that immune checkpoint inhibitors (ICIs) yield modest objective response rates in advanced sarcomas, approximately 10–15% across unselected populations, yet subset analyses suggest superior outcomes in tumours characterised by TLS, high B-cell signatures, and an immune-active microenvironment [63].

In this context, patients with limited metastatic burden, minimal tumour-driven immunosuppression, and favourable immune biomarkers might represent an ideal niche for combining metastasis-directed therapy (surgery, SBRT, ablation) with ICIs. The biological rationale is compelling: local ablative treatments can induce immunogenic cell death, promote the release of tumour antigens, and augment systemic immune surveillance, an “in situ vaccination” effect that is recognised in polymetastatic disease but may be even more effective when the residual disease burden is low [64,65,66,67].

Importantly, recent prospective data now strengthen the translational rationale for integrating ICIs into multimodal STS management. A randomised, open-label trial (NCT03092323, identifier code assigned in www.clinicaltrials.gov registry, last accessed on 28 November 2025.) evaluated the addition of pembrolizumab to preoperative radiotherapy and surgery in patients with localised, high-risk grade 2–3 undifferentiated pleomorphic sarcoma or pleomorphic/dedifferentiated liposarcoma of the extremity. In this study, 127 patients (median follow-up 43 months) were analysed in the modified intention-to-treat cohort: the pembrolizumab-radiotherapy–surgery strategy significantly improved disease-free survival compared with radiotherapy and surgery alone (HR 0.61, 90% CI 0.39–0.96; one-sided *p* = 0.035), with a 15% absolute increase in 2-year DFS (67% vs. 52%). Although grade ≥ 3 events were more frequent in the experimental arm (56% vs. 31%), the study establishes the first high-level evidence that immune–radiotherapy combinations can improve clinically meaningful endpoints in high-risk STS [68].

While the trial focused on localised disease, its findings provide a mechanistic and clinical foundation for exploring similar multimodal strategies in the oligometastatic setting, where disease burden is lower, immune suppression is reduced, and ablative therapy may act synergistically with ICIs to eliminate micrometastatic clones. Further clinical studies are currently underway to investigate the synergistic interaction between SBRT and immunotherapy in oligometastatic STSs (Table 1).

Beyond immunotherapy, vaccine-based approaches and adoptive T-cell strategies targeting sarcoma-specific antigens (e.g., NY-ESO-1 in synovial sarcoma, MAGE antigens) are under investigation and may be most rational in low-burden oligometastatic settings, where tumour antigen heterogeneity is reduced and micro-metastatic disease may be more immunologically visible [69]. Furthermore, epigenetic or targeted therapies (such as HDAC or EZH2 inhibitors) may enhance tumour immunogenicity and synergise with ICIs and local therapy—particularly when used to treat minimal residual disease after ablative intervention [70,71].

Collectively, this biological and clinical framework supports the design of prospective trials in oligometastatic STS that integrate molecular/immune stratification (e.g., TLS status, CINSARC, ctDNA), local ablative therapy, and immune/targeted combinations. Such studies have the potential to shift the therapeutic intent from palliative control toward durable remission or cure.

## 8. Decision-Making Framework for Identifying Oligometastatic STS Candidates for Metastasis-Directed Therapy

In patients presenting with metastatic intermediate- or high-grade STS, a structured clinical–biological assessment can help differentiate true oligometastatic biology from early polymetastatic evolution and guide the appropriate use of local definitive therapies (metastasectomy, SBRT, ablation). In our view, the decision-making process should integrate those clinical and biological factors most likely to indicate an oligometastatic phenotype in which DLTs may achieve durable disease control, allow deferral of systemic therapy, or—following metachronous oligo-recurrence—support iterative local treatment approaches.

Priority factors suggesting a true oligometastatic phenotype:

- Low metastatic burden: typically ≤3–5 lesions confined to a single organ, most commonly the lung.
- Favourable prognostic estimates on validated clinical nomograms (Sarculator, MSKCC, PERSARC).
- Long disease-free interval preceding the first metastatic event (>12–18 months).
- Histologic subtypes known for relatively indolent or site-restricted metastatic behaviour (e.g., myxoid liposarcoma, synovial sarcoma with isolated pulmonary metastases).

Secondary or investigational factors (supportive but not yet validated for routine selection):

- Low-risk molecular features: CINSARC-low status, low genomic complexity or tumour mutational burden, and a B-cell/TLS-rich immune microenvironment.
- Low or undetectable ctDNA at baseline or post-DLT, consistent with limited systemic tumour burden and reduced metastatic kinetics.
- Immune-activation features: high CD8^+^ T-cell infiltration, increased T-cell clonality, or composite immune-activation signatures beyond TLS.

This integrated framework can assist multidisciplinary teams in distinguishing pseudo-oligometastatic presentations from genuinely limited metastatic trajectories, thereby refining patient selection for metastasis-directed interventions and supporting rational postponement of systemic therapy when clinically appropriate.

## 9. Artificial Intelligence-Driven Prediction of Oligometastatic Behaviour in STS

The increasing availability of high-dimensional molecular, imaging, and clinical data has created a fertile ground for the application of artificial intelligence (AI) and machinelearning (ML) approaches in oncology. Within this context, the prediction of oligometastatic versus polymetastatic evolution represents a paradigmatic challenge well-suited to computational genomics and bioinformatics. Oligometastatic disease is not defined solely by metastatic count or anatomical distribution, but rather reflects an underlying biological state governed by tumour-intrinsic programmes, immune–microenvironmental interactions, and evolutionary dynamics. AI-based models offer a unique opportunity to integrate these heterogeneous data layers into unified predictive frameworks.

From a genomic perspective, ML algorithms have already demonstrated their capacity to extract prognostically meaningful patterns from transcriptomic and genomic datasets in sarcomas. Supervised learning approaches have been successfully applied to gene-expression signatures related to chromosomal instability, cell-cycle regulation, and immune activation, enabling refined stratification of metastatic risk beyond conventional clinicopathologic variables [72,73]. Extending these models specifically to discriminate oligometastatic from polymetastatic trajectories is a logical next step, particularly when genomic complexity metrics (such as CINSARC-related features), copy-number burden, and mutational profiles are jointly analysed using ensemble or deep-learning architectures.

Radiomics and AI-based analysis of cross-sectional imaging represent another rapidly evolving domain. Quantitative imaging features extracted from CT and PET scans—capturing tumour shape, texture, heterogeneity, and growth kinetics—have been shown to correlate with metastatic potential, response, and survival across multiple cancers. In soft-tissue sarcomas, AI-driven radiomics could help distinguish true oligometastatic disease from pseudo-oligometastatic presentations that conceal aggressive systemic biology, especially when longitudinal imaging data are incorporated into temporal ML models [62]. Liquid-biopsy data further enrich AI-based prediction. Circulating tumour DNA (ctDNA) provides a dynamic, patient-specific measure of tumour burden and clonal evolution. ML models integrating ctDNA allele frequencies, mutation patterns, and clearance kinetics after local therapy have shown strong predictive value for early relapse and metastatic progression in solid tumours. In sarcomas, combining ctDNA features with genomic and immune variables through AI pipelines could enable real-time classification of metastatic state, identifying patients most likely to sustain long-term control after local ablative interventions [74].

Critically, the immune microenvironment is increasingly recognised as a determinant of metastatic competence. AI-based deconvolution of bulk RNA-sequencing data and digital pathology analyses can quantify immune-cell populations, tertiary lymphoid structures, and spatial immune organisation with high precision. Computational immune profiling has already been associated with survival and immunotherapy response in sarcomas [75,76,77]. Integrating these immune features into multimodal AI models may improve discrimination between oligometastatic and polymetastatic biology, particularly when immune activation counterbalances otherwise aggressive genomic features.

Ultimately, the greatest promise of AI lies in multimodal integration. Advanced ML frameworks—such as graph neural networks and deep multimodal learning—can simultaneously process clinical variables, genomics, transcriptomics, radiomics, immune metrics, and ctDNA data to generate individualised predictions of metastatic evolution [78]. Such models align directly with the goals of computational cancer genomics: transforming complex biological data into actionable clinical insights. In the context of intermediate/high-grade soft-tissue sarcomas, AI-driven prediction of oligometastatic behaviour could fundamentally reshape clinical decision-making. Rather than relying on static lesion counts, clinicians could deploy biologically informed algorithms to guide the selection of metastasis-directed therapies, tailor surveillance intensity, and optimise integration with systemic or immune-based treatments. Prospective validation of AI tools within biomarker-stratified clinical trials represents a critical research priority and a natural convergence point for computational genomics, bioinformatics, and precision oncology.

## 10. Future Directions and Clinical Research Priorities

The recognition of an oligometastatic phenotype within intermediate/high-grade STS has profound implications for clinical trial design, biomarker development and multidisciplinary practice. At present, evidence for metastasis-directed local therapies is overwhelmingly retrospective and confounded by selection bias. To address this, future trials should adopt biomarker-enriched designs, stratifying or randomising patients based on validated molecular and immune classifiers (e.g., CINSARC low-risk, TLS positive, ctDNA negative) to evaluate the incremental benefit of local therapy ± systemic/immune adjuncts. Moreover, a new paradigm of “molecular minimal disease” surveillance may emerge: for example, patients who achieve molecular remission (undetectable ctDNA) after metastasis-directed therapy might safely enter a surveillance-dominated pathway, while those with persistently detectable ctDNA might be intuitively directed to early systemic therapy or inclusion in interventional trials. To operationalise these strategies, collaboration among high-volume sarcoma centres, centralised biobanks and federated registries is essential, enabling standardised collection of imaging, pathology, immune profiling and liquid-biopsy data. In parallel, translational sub-studies embedded within prospective trials should interrogate the effects of local ablative therapy on systemic tumour evolution and immune responses, using sequential biopsies, ctDNA kinetics and immune-profiling pre- and post-intervention. From a clinical implementation standpoint, decision algorithms need refinement: defining the optimal number of lesions, organ-involvement threshold, timing of systemic therapy and choice of ablative modality remains unresolved.

Complete standardisation across centres is challenging—particularly for liquid biopsy—because pre-analytical variables (tube type, time-to-processing, centrifugation steps, storage conditions) and analytical workflows (assay chemistry, depth, bioinformatics pipelines) can introduce measurable variability. However, in our view, meaningful standardisation is still achievable at the level that matters for operationalising biomarker-guided strategies, by adopting a tiered harmonisation framework: (i) a mandatory “core” SOP for the most variance-driving steps (e.g., plasma collection in cfDNA-stabilising tubes, defined processing time windows, double-spin protocols, uniform storage temperature and freeze–thaw limits) and (ii) recommended/optional elements that may differ by site but are fully captured in structured metadata. This approach allows multicentre studies to control the dominant sources of technical noise while remaining feasible in real-world sarcoma networks. Importantly, partial standardisation can still support implementable clinical strategies, provided that (a) a minimum technical baseline is enforced, (b) harmonised reporting is used (e.g., tumour fraction/variant allele fraction, limit of detection, QC thresholds), and (c) prospective studies include bridging/calibration procedures (centralised reference materials, inter-laboratory ring trials, and/or central bioinformatics processing). In practice, many decision frameworks do not require absolute cross-platform equivalence; they can rely on within-patient kinetics (clearance vs. persistence/rise) and threshold-based categories (detectable vs. undetectable; low vs. high) that are more robust to residual inter-site variation than raw quantitative values. Where heterogeneity remains unavoidable, contemporary statistical harmonisation (batch-effect modelling) and sensitivity analyses can further mitigate bias, but we agree that rigorous SOPs for key pre-analytical steps and transparent metadata are essential to avoid confounding and to enable reproducible, biomarker-stratified trials in oligometastatic STS.

Ultimately, the goal is to shift from a “one-size-fits-all” palliative mindset toward a biologically guided, precision-oncology paradigm in which oligometastatic STS are managed with curative intent, combining high-technology local therapy, immune and molecular stratification, and vigilant surveillance. High-technology strategies—such as comprehensive molecular profiling, AI-driven multimodal classifiers, advanced SBRT platforms, and serial ctDNA monitoring—may currently be more readily implemented in well-resourced healthcare systems. However, although our long-term vision is a biologically guided precision-oncology model, several lower-cost and widely accessible strategies can already help move away from a purely palliative mindset, even in resource-constrained settings. First, careful clinical selection remains a powerful and low-cost tool. Readily available parameters—such as number of metastases (≤3–5), confinement to a single organ (most commonly lung), long disease-free interval (>12–18 months), good performance status, and favourable histology—can be integrated into structured decision algorithms without the need for advanced molecular assays. Second, conventional imaging-based surveillance, when applied systematically and at appropriate intervals, can support the early detection of limited metastatic relapse. High-quality CT-based follow-up interpreted within a multidisciplinary sarcoma board allows timely identification of patients eligible for metastasis-directed therapy before widespread dissemination occurs. Third, surgery remains the cornerstone of metastasis-directed treatment in many settings and is often more accessible than advanced radiation technologies. Pulmonary metastasectomy, when feasible, can be performed in centres with thoracic surgical expertise without requiring sophisticated infrastructure beyond standard oncologic care. Similarly, selected ablative procedures (e.g., radiofrequency ablation) may be implemented in interventional radiology units already available in many tertiary hospitals. Finally, a conceptual shift is itself low-cost. Moving from an automatic “systemic therapy only” approach to a structured evaluation of curative-intent options—based on clinical criteria and multidisciplinary discussion—does not require advanced genomic testing but rather an organisational and cultural reorientation. Even incremental implementation of these strategies can partially transition care from a strictly palliative framework toward a risk-adapted, potentially durable-control paradigm.

## Figures and Tables

**Table 1:** Trials exploring immune–radiotherapy synergy in oligometastatic STS.

Trial	NCT Registry	Phase	Population/Oligometastatic Eligibility	Intervention (Immune Agent + Local Therapy)	Primary Endpoint
STEREOSARC—randomised SBRT ± immunotherapy in oligometastatic STS	NCT03548428 (STEREOSARC)	Phase II (randomised)	Adults with oligometastatic STSs (multisite oligometastases); predefined ≤ number lesions per protocol	Multisite SBRT ± anti-PD-L1 (atezolizumab) concomitant therapy (ICI + SBRT arm vs. SBRT alone)	PFS
Pembrolizumab + SBRT (pilot/early phase)—sarcoma cohort	NCT05488366	Phase I	Advanced/metastatic or recurrent STSs (includes patients amenable to SBRT; oligometastatic subsets eligible)	Pembrolizumab (anti-PD-1) combined with multisite SBRT (safety/feasibility)	Safety/feasibility; preliminary anti-tumour activity
Single-arm SBRT in metastatic sarcoma (radiotherapy-focused)—small multicohort	NCT05821231	Phase I	Metastatic STSs with lung metastases (small cohorts; potential oligometastatic enrolment)	SBRT to lung metastases (protocol includes radiotherapy endpoints; may be combined with systemic agents per cohort) + bispecific antibody anti PD-1/CTLA-4 (MEDI5752 *)	Safety/local control

CTLA-4: Cytotoxic T-lymphocyte antigen 4; ICI: Immune checkpoint inhibitor; PD-1: Programmed cell death protein 1; PD-L1: Programmed death-ligand 1; PFS: Progression-free survival; SBRT: Stereotactic body radiotherapy; STS: Soft-tissue sarcoma. NCT03548428, NCT05488366, and NCT05821231 are identifier codes assigned in www.clinicaltrials.gov registry, last accessed on 28 November 2025. * MEDI5752 is the development code assigned by the pharmaceutical company.

## Data Availability

No new data were created in this work.

## Abbreviations

The following abbreviations are used in this manuscript:

STS	Soft-tissue sarcomas
FNCLCC	French Federation of Cancer Centers Sarcoma Group
NCI	National Cancer Institute
ASIR	Age-standardised incidence rate
NED	No evidence of disease
SBRT	Stereotactic body radiotherapy
CINSARC	Complexity INdex in SARComas
TLS	Tertiary lymphoid structures
PD-1	Programmed cell death protein 1
ctDNA	Circulating tumour DNA
MRD	Minimal residual disease
TMB	Tumour mutational burden
PD-L1	Programmed death-ligand 1
UPS	Undifferentiated pleomorphic sarcoma
AJCC	American Joint Committee on Cancer
MSKCC	Memorial Sloan Kettering Cancer Center
PERSARC	Personalised Sarcoma Care
PFS	Progression-free survival
OS	Overall survival
DFI	Disease-free interval
SABR	Stereotactic ablative radiotherapy
TME	Tumour microenvironment
ICI	Immune checkpoint inhibitor
ICIs	Immune checkpoint inhibitors
CTLA-4	Cytotoxic T-lymphocyte antigen 4
DLTs	Definitive local therapies
AI	Artificial intelligence
ML	Machine learning
CT	Computed tomography
PET	Positron emission tomography
RNA-seq	RNA sequencing
